# Duration of rectal colonization with extended-spectrum beta-lactamase-producing *Escherichia coli*: results of an open, dynamic cohort study in Dutch nursing home residents (2013–2019)

**DOI:** 10.1186/s13756-022-01132-9

**Published:** 2022-07-15

**Authors:** Veronica Weterings, Wouter van den Bijllaardt, Martin Bootsma, Yvonne Hendriks, Linda Kilsdonk, Ans Mulders, Jan Kluytmans

**Affiliations:** 1 Department of Infection Control, Amphia Hospital, P.O. Box 90158, 4800 RK Breda, The Netherlands; 2 Microvida Laboratory for Microbiology, Amphia Hospital, P.O. Box 90158, 4800 RK Breda, The Netherlands; 3 Julius Center for Health Sciences and Primary Care, UMC Utrecht, Utrecht University, P.O. Box 85500, 3508 GA Utrecht, The Netherlands; 4 Thebe Long-Care Facilities, P.O. Box 9757, 4801 LW Breda, The Netherlands

**Keywords:** ST131, ESBL, Colonization, *E. coli*, Survival curve

## Abstract

**Background:**

In 2016, a study in a Dutch nursing home showed prolonged colonization duration of extended-spectrum *β*-lactamase-producing (ESBL)-ST131 compared to ESBL-non-ST131. In this study, we assessed the duration of rectal ESBL-producing *E. coli* (ESBL-EC) colonization in residents in the same nursing home for an extended period of six years. We aimed to estimate the influence of a possible bias when follow up is started during an outbreak.

**Methods:**

Between 2013 and 2019, repetitive point prevalence surveys were performed by culturing rectal or faecal swabs from all residents. Kaplan–Meier survival analysis was performed to calculate the median time to clearance of ESBL-EC with a log-rank analysis to test for differences between ESBL-ST131 and ESBL-non-ST131.

**Results:**

The study showed a median time to clearance of 13.0 months (95% CI 0.0–27.9) for ESBL-ST131 compared to 11.2 months (95% CI 4.8–17.6) for ESBL-non-ST131 (*p* = 0.044). In the subgroup analysis of residents who were ESBL-EC positive in their first survey, the median time to clearance for ST131 was 59.7 months (95% CI 23.7–95.6) compared to 16.2 months (95% CI 2.1–30.4) for ESBL-non-ST131 (*p* = 0.036). In the subgroup analysis of residents who acquired ESBL-EC, the median time to clearance for ST131 was 7.2 months (95% CI 2.1–12.2) compared to 7.9 months (95% CI 0.0–18.3) for ESBL-non-ST131 (*p* = 0.718). The median time to clearance in the ESBL-ST131 group was significantly longer in residents who were ESBL-ST131 colonised upon entering the study than in residents who acquired ESBL-ST131 during the study (*p* = 0.001).

**Conclusion:**

A prolonged colonization with ESBL-ST131 was only found in the subgroup who was ESBL-EC positive upon entering the study. The prolonged duration with ESBL-ST131 in the previous study was probably biased by factors that occured during (the start of) the outbreak.

**Supplementary Information:**

The online version contains supplementary material available at 10.1186/s13756-022-01132-9.

## Background

*Escherichia coli* (*E. coli*) sequence type (ST) 131 is an extraintestinal pathogenic *E. coli* (ExPEC) [1, 2], and is nowadays the predominant *E. coli* lineage among ExPEC isolates worldwide [3]. Moreover, ST131 is associated with the worldwide spread of the CTX-M-15 extended spectrum *β*-lactamase (ESBL) resistance gene [3]). ESBL-producing ST131 (ESBL-ST131) is a major contributor to hospital- and community-acquired infections, such as urinary tract infections and bloodstream infections [4, 5]. ESBL-ST131 infections are most common among elderly people and ESBL-ST131 carriage is particularly prevalent in nursing homes and long-term care facilities [6, 7].

Despite many studies examining the epidemiology of ESBL-ST131, the reason why this clone is so successful is still not fully understood. In 2016, Overdevest et al. evaluated the duration of colonization with ESBL-ST131 in residents of a Dutch nursing home where ST131 had spread extensively [8]. Between March 2013 and April 2014, six point prevalence surveys were performed at intervals of three months by culturing faeces or rectal swabs. The study showed a prolonged colonization duration of residents with ESBL-ST131, with a median time to clearance of 13 months compared to two to three months for other ESBL-producing *E. coli* (ESBL-non-ST131) (*p* < 0.001).

Overdevest et al. started the study after the detection of an ESBL-ST131 outbreak. They calculated the duration of colonization from the first positive culture within the study period. With this approach, ~ 50% of the cases were already colonized at the first screening (onset date of colonization unknown). This approach (outbreak setting and unknown onset date) may introduce bias as it is more likely to start the observation period of a case shortly after the time of acquisition for ESBL-ST131 compared to ESBL-non-ST131. Unknown factors (e.g. resident’s characteristics, institutional characteristics and/or environmental factors) may have contributed to the difference observed in duration of colonization. The point prevalence surveys in the Dutch nursing home of the aforementioned study were continued until June 2019. In this study, we evaluated the duration of rectal ESBL-EC colonization of residents in the same nursing home over an extended period of 6 years (March 2013 to June 2019).

The objective of this study was to compare the duration of rectal ESBL-EC colonization for ESBL-ST131 versus ESBL-non-ST131. In addition to the overall comparison, we performed a comparison between residents who acquired ESBL-EC during the study and those who were colonized upon entering the study. We aimed to estimate the influence of a possible bias when follow up is started during an outbreak.

## Methods

### Detection of ST131 outbreak and setting

**Fig. 1:**
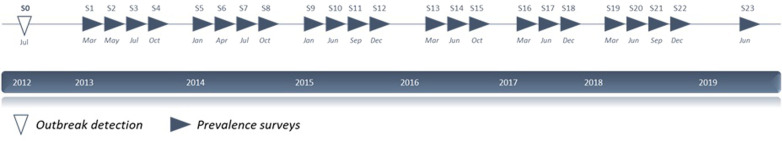
Overview of the outbreak detection and subsequent prevalence studies in the nursing home

As part of the standard infection control policy, a prevalence survey was performed in a Dutch nursing home (Fig. 1) in 2012, showing a high prevalence (20.6%) of rectal ESBL colonization [7]. Strain typing showed the extensive presence of ESBL-ST131, among smaller clusters and unique strains of other sequences types. Outbreak containment measures were implemented, including repetitive prevalence surveys at intervals of three to six months by culturing faeces or rectal swabs from all residents. In June 2019, the prevalence surveys were ended.

### Study design and study population

We conducted an open, dynamic cohort study based on data generated by repeated point prevalence surveys from March 2013 to June 2019 as part of the existing infection control policy in a nursing home in the Netherlands. Residents with at least one ESBL-EC positive rectal or faecal swab obtained in a prevalence survey during the study period were eligible for inclusion. Residents acquiring ESBL-EC in the final prevalence survey (June 2019) were not included.

### Detection of ESBL-producing E. coli

The intended sampling scheme consisted of a quarterly screening of all residents of the wards involved. Throughout all point prevalence surveys colonization of ESBL-EC was determined by culture of rectal or faecal samples collected using Eswab (Copan, Italy). Swabs were inoculated directly on extended-spectrum β-lactamase screening agar (EbSA) (AlphaOmega,’s-Gravenhage, Netherlands) and 5% Sheep blood agar (growth control). The remaining Eswab fluid was transferred in selective enrichment broth consisting of 5 mL tryptic soy broth containing cefotaxime (0.25 mg/L) and vancomycin (8 mg/L) (TSB-VC). After 18–24 h of incubation (35–37 °C), the TSB-VC was subcultured on an EbSA plate. For all Gram-negative rods growing on the EbSA, either directly or after overnight culture in enrichment broth, species identification and susceptibility testing was performed by MALDI-TOF (bioMérieux, Marcy l’Etoile, France) and VITEK 2 (bioMérieux, Marcy l’Etoile, France), respectively. Phenotypic ESBL production was confirmed by double disk method [9].

### Genotyping and strain typing

All phenotypically confirmed ESBL-EC underwent an O25:ST131-specific PCR [10]. ESBL genotyping was performed using a micro-array (CheckPoints, Wageningen, the Netherlands) [11, 12] and strain typing by using amplified fragment length polymorphism (AFLP) [13]. An AFLP cluster was assigned based on both visual and computerised interpretation of AFLP patterns.

ESBL genotyping and strain typing were performed for the first ESBL-EC from each resident and for subsequent ESBL-EC strains that were not similar to the first strain. Similarity was defined as identical species and O25:ST131 status and absence of major differences in susceptibility (susceptible vs. resistant) for all antibiotics tested.

### Definitions

Rectal colonization with ESBL-EC was defined as detection of ESBL-EC in at least one rectal or faecal swab. Acquisition of ESBL-EC was defined as a resident with at least one ESBL-EC negative culture before the first ESBL-EC positive culture. A resident was considered no longer colonized (loss of colonization) when, after a previous ESBL-EC positive culture, at least one rectal or faecal swab did not reveal ESBL-EC or when strain typing showed a different MLST or cluster type than found in the previous ESBL-EC positive culture of the resident (Additional file 1: Fig. 1). Residents were only included in the study once, i.e. the first time ESBL-EC carriage was detected in the study period.

Additional analyses were performed whereby loss of colonization status was reached when at least two (instead of one) rectal or faecal swabs no longer yielded ESBL-EC or when two swabs showed a different sequence type and/or cluster type than found in the previous ESBL-EC positive culture of the resident (Additional file 2: Fig. 2).

### Variables

Data concerning gender, date of birth, and if applicable date of discharge from nursing home or date of death were obtained from the nursing homes records.

### Duration of colonization

For each resident, the duration of rectal colonization with ESBL-EC was calculated as the time from the first ESBL-EC positive sample until the last ESBL-EC positive sample plus half the time between the last ESBL-EC positive culture and the first ESBL-EC negative culture (Additional file 1: Fig. 1a) or the first ESBL-EC positive culture with a different cluster/sequence type (Additional file 1: Fig. 1b). Both situations were labelled as an ‘event’ in the Kaplan–Meier survival analysis.

In residents whose last sample was still ESBL-EC positive (no loss of colonization) at the end of the study period, the time of rectal colonization of ESBL-EC was calculated as the time from the first ESBL-EC positive sample until the last ESBL-EC positive sample (Additional file 1: Fig. 1c). For residents who died or were discharged before the end of the study, the time of rectal colonization of ESBL-EC was calculated as the time from the first ESBL-EC positive sample until time of death or discharge (Additional file 1: Fig. 1d). These cases were labelled as ‘censored’ in the Kaplan–Meier survival analysis.

### Statistical analysis

Data analysis was performed using statistical package for social science (SPSS) version 25. The prevalence of carriage was calculated as the percentage of carriers among all residents. Comparisons of categorical variables between the ESBL-ST131 and ESBL-non-ST131 group were performed with Fisher’s exact test and continuous variables were compared by the Mann–Whitney *U* test. Kaplan–Meier survival analysis was performed to calculate the median time to clearance for ESBL-EC with a Log-Rank analysis to test for differences between ESBL-ST131 and ESBL-non-ST131 and between ESBL-ST131 positive at start and ESBL-ST131 acquisition during study. A p-value < 0.05 was considered as statistically significant.

## Results

**Fig. 2:**
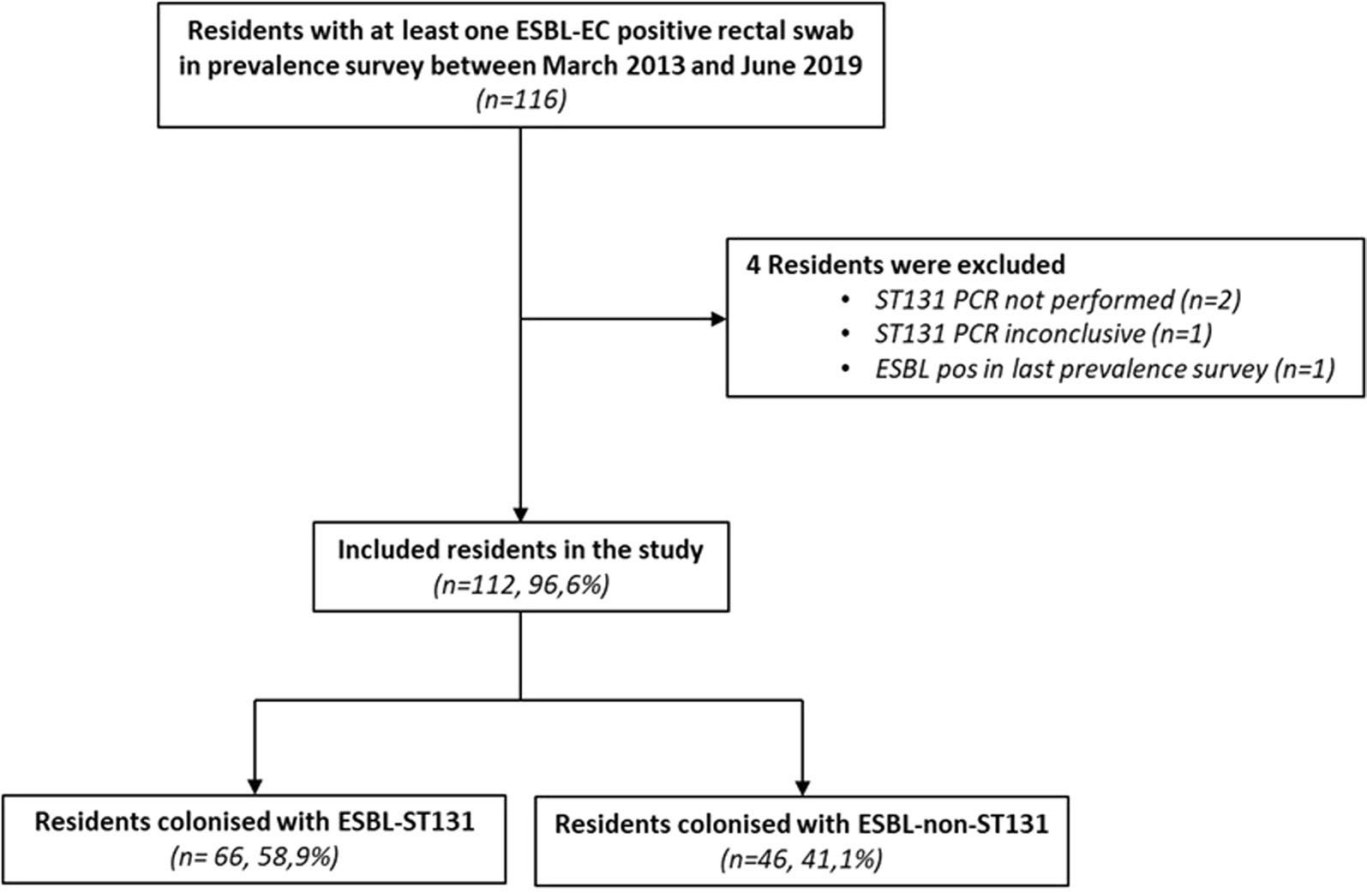
Flowchart for inclusion

Between March 2013 and June 2019, 23-point prevalence surveys were performed at intervals of three to six months (Fig. 1). A total of 4.980 samples were cultured from 1.806 unique residents. In total, 116 residents (6.4%) had at least one ESBL-EC positive sample in any prevalence survey (Fig. 2). Of these, four residents were excluded from the study because O25:ST131-specific PCR was not performed (*n* = 2), the result of the O25:ST131-specific PCR was inconclusive (*n* = 1) or the resident acquired ESBL-EC in the final prevalence survey (*n* = 1).

Therefore, 112 residents were included: 66 residents (58.9%) with ESBL-ST131 rectal colonization and 46 residents (41.1%) with ESBL-non-ST131 rectal colonization (Fig. 2). The median age on the day of the first ESBL-EC positive rectal sample was 82 years (interquartile range (IQR): 75–88) and 63/112 (56.3%) of the residents were female. The median follow-up time (period between first positive ESBL-EC culture and last culture, regardless of culture result of the last culture) was 17.9 months (IQR: 6.0–40.6) for ESBL-ST131 and 10,1 months (IQR: 2,8–30,2) for ESBL-non-ST131 (p = 0.106).

In total 55 of 112 residents (49.1%) were already colonized with ESBL-EC at the time they first participated in any prevalence survey: 33/66 (50.0%) for ESBL-ST131 and 22/46 (47.8%) for ESBL-non-ST131 (*p* = 0.850)., Of these 55, 32 (58.2%) were colonized in the first prevalence survey in March 2013 (ESBL-ST131: 23/32 (71.9%); ESBL-non-ST131: 9/32 (28.1%)). Furthermore, 49/112 (43.8%) were newly admitted to the nursing home after the first prevalence survey in March 2013. The age at time of first ESBL-EC positive sample and gender for ESBL-ST131 and ESBL-non-ST131 per analysis are presented in Table 1.

**Table 1:** Age on the day of the first ESBL-EC positive rectal sample and gender characteristics of the residents for ESBL-ST131 and ESBL-non-ST131, per analysis

MLST group	All residents with at least one positive ESBL-EC culture (*n* = 112)		Residents who were ESBL positive in their first prevalence survey (n = 55)		Residents who acquired ESBL-EC during the study (n = 57)	
	ESBL-ST131 (*n* = 66)	ESBL-non-ST131 (*n* = 46)	ESBL-ST131 (*n* = 33)	ESBL-non-ST131 (*n* = 22)	ESBL-ST131 (*n* = 33)	ESBL-non-ST131 (*n* = 24)
Age in years, median (IQR)	82 (76–88)	82 (74 –87)	83 (74–88)	84 (76–87)	82 (77.5–88.5)	81 (73.0–86.5)
Female gender, *n* (%)	37 (56.0%)	26 (56.5%)	19 (57.6%)	11 (50.0%)	18 (54.5%)	15 (62.5%)

### Molecular characterization of ESBL genes

The predominant ESBL genotype in the ESBL-ST131 group was *bla*CTX-M-15 (n = 57; 86.4%), followed by *bla*CTX-M-9 (n = 9; 13.6%). The ESBL genes in the non-ESBL-ST131 group were more diverse, the predominant ESBL genotype was *bla*CTX-M-9 (n = 13; 28.3%), followed by *bla*CTX-M-15 (n = 11; 23.9%), *bla*CTX-M-1 (n = 8; 17.4%), *bla*CTX-M-3 (n = 8; 17.4%), *bla*TEM (n = 3; 6.5%) and *bla*SHV (n = 3; 6.5%).

### Duration of colonization

During the study period loss of colonization of ESBL-EC (based on one ESBL-EC negative swab) or colonization with a different cluster type than in previous cultures (based on one swab with a different cluster type) was observed in 31/66 ESBL-ST131 carriers (47.0%) and in 26/46 ESBL-non-ST131 carriers (56.5%) (*p* = 0.343). Three of 112 residents (2.7%) with an ESBL-negative sample became ESBL-positive again in the sample following the first ESBL-EC negative sample. An overview of the proportion and reasons for events or censoring for the Kaplan–Meier survival analyses per selection group is shown in Additional file 6: Tables 1–3.

An overview of the proportion and reasons for events or censoring for the Kaplan–Meier survival analyses per selection group based on at least two (instead of one) rectal or faecal swabs is shown in Additional file 6: Tables 4–6.

**Fig. 3:**
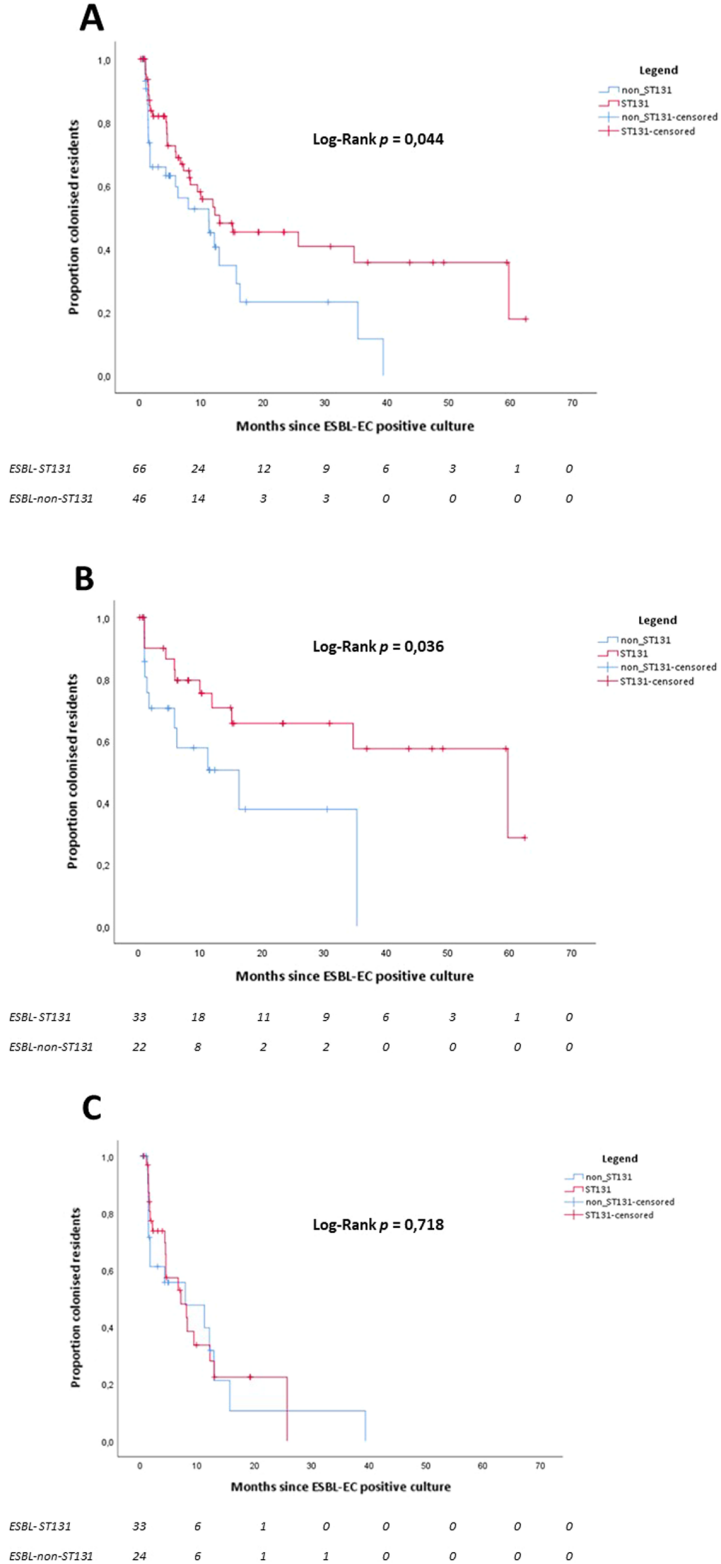
Kaplan–Meier curves of survival probability of residents colonised with ESBL-producing *E. coli*, by ST131 and non-ST131 for all residents (**A**), for residents who were ESBL-EC positive in their first prevalence survey in the study (**B**), and for residents who acquired ESBL-EC during the study (**C**). Values below the survival graphs indicate the number of residents per MLST group

Figure 3A shows the Kaplan–Meier survival curve of ESBL-EC colonization over time for all included residents. The median time to clearance for ESBL-ST131 was 13.0 months (95% CI 0.0–27.9) compared to 11.2 months (95% CI 4.8–17.6) for ESBL-non-ST131 (*p* = 0.044). Figure 3B shows the Kaplan–Meier survival curve of ESBL-EC colonization over time for residents who were ESBL-EC positive in their first prevalence survey in the study. The median time to clearance for ESBL-ST131 was 59.7 months (95% CI 23.7–95.6) compared to 16.2 months (95% CI 2.1–30.4) for ESBL-non-ST131 (*p* = 0.036). Figure 3C shows the Kaplan–Meier survival curves of ESBL-EC colonization over time for residents who acquired ESBL-EC during the study. The median time to clearance for ESBL-ST131 was 7.2 months (95% CI 2.1–12.2) compared to 7.9 months (95% CI 0.0–18.3) for ESBL-non-ST131 (*p* = 0.718).

**Fig. 4:**
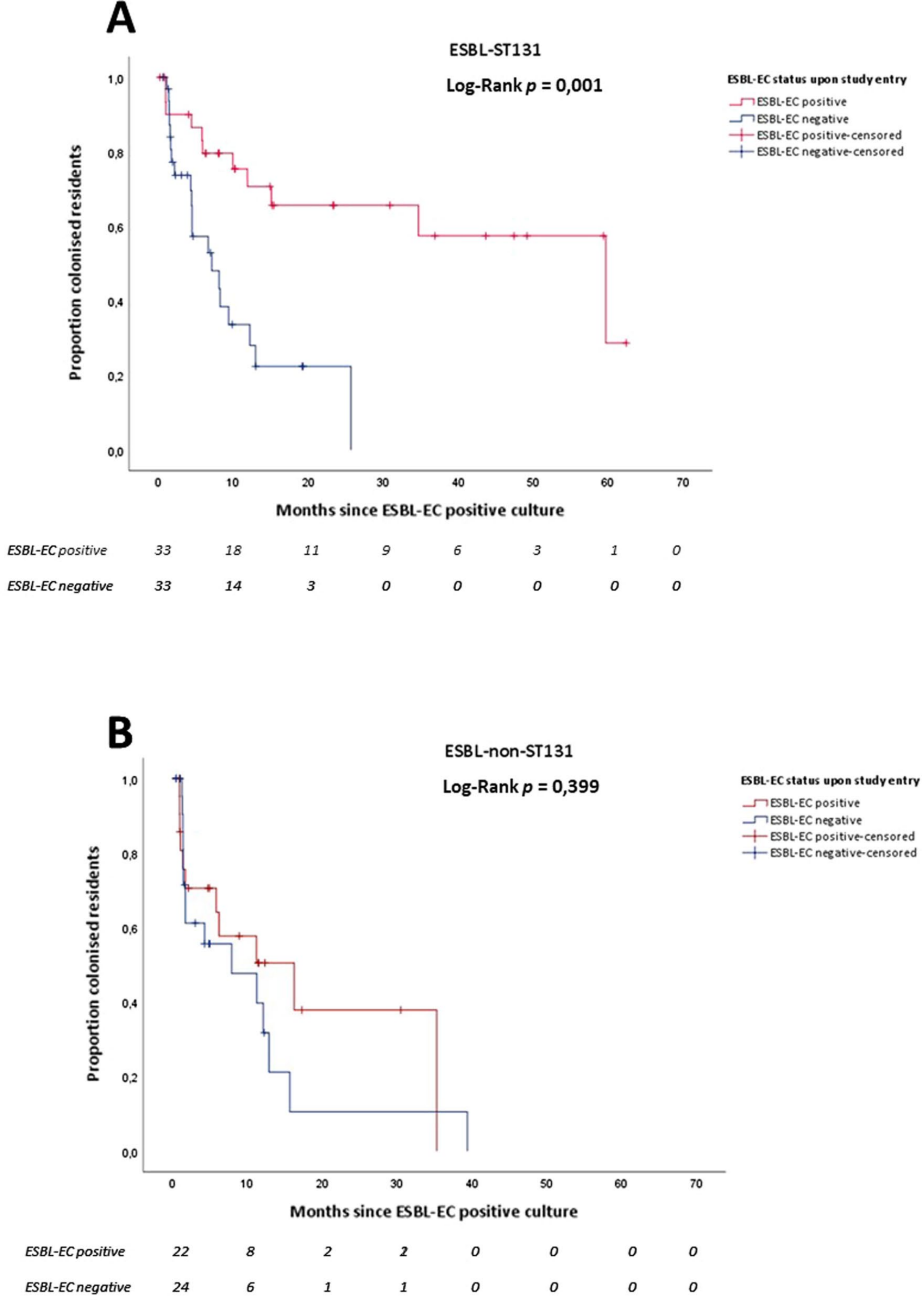
Kaplan–Meier survival curve of survival probability of residents colonised with ESBL-ST131 (**A**) and ESBL-non-ST131 (**B**) by ESBL-EC status (positive or negative) upon entry of the study

Kaplan–Meier curves of survival probability of residents colonised with ESBL-producing *E. coli*, by ST131 and non-ST131 with two (instead of one) ESBL-EC negative cultures/other strain type to consider a resident no longer colonised are shown in the Additional file 2–5: Figs. 2–5. In addition, no significant difference was found between the median time to clearance for ESBL-ST131 and ESBL-non-ST131.

To further investigate the influence of ESBL-EC colonization status upon inclusion in the study (meaning ESBL-EC positive in first prevalence survey versus acquisition during study period), an additional Kaplan–Meier survival analysis was performed. In this analysis we first stratified ESBL-ST131 and ESBL-non-ST131. Within these two groups we compared the patients who were positive in the first culture and those who had at least one negative before the first positive culture. In the ESBL-ST131 group, the median time to clearance was significantly longer in residents who were ESBL-EC colonised upon entering the study than in residents who acquired ESBL-EC (*p* = 0.001) (Fig. 4A). In the subgroup ESBL-non-ST131 no significant differences were found between the two groups (*p* = 0.399) (Fig. 4B).

## Discussion

In this study, the median time to clearance for ESBL-ST131 was significant longer for residents who were already ESBL-EC colonised upon entry in the study, whereas this difference was not observed in residents who acquired ESBL-EC during the follow up period. This difference was not found for other sequence types.

We evaluated the median time to clearance of rectal ESBL- EC colonization in anursing home over a period of six years in all residents, and performed a subgroup analysis for residents who were already ESBL-EC colonised upon study entry and compared it to those who acquired ESBL-EC during their stay at the nursing home. In the primary analysis (all residents) we found a significant prolonged median time to clearance for ESBL-ST131. For residents who were ESBL-EC positive upon study entry this difference was much more pronounced, while no difference was observed for residents who acquired ESBL-EC during the study.

A further analysis was done based on a stratification of the ESBL-ST131 and ESBL-non-ST131 groups. Per group we compared those who were already colonised in the first culture with those who acquired ESBL-EC during the follow-up period. This analysis revealed a significantly longer time to clearance in residents who were colonised with ESBL-ST131 upon entry in the study compared to those who acquired ESBL-ST131 during the study. This difference was not found for other sequence types. These results indicate that the prolonged duration of colonization with ESBL-ST131 found in the initial study in 2016 [8], was caused by the residents who were already colonized with ESBL-ST131 when the follow-up was started. It is not clear what the underlying reasons are for this phenomenon. A possible explanation could be that the residents who were colonized with ESBL-ST131 during the outbreak period, were more susceptible to long-term colonization and that this group was overrepresented. However, many new residents entered the cohort in the following years and some acquired ESBL-ST131. If there was a group of individuals who were more likely to be colonized for longer periods, there must have been some of those in the group with acquired ESBL-ST131 as well. This was not the case and we consider this explanation unlikely. Another explanation for the longer colonization but also for the observed higher mortality in the ESBL-ST131 group that was colonized upon study entry could be that this prolonged colonization was limited to a subgroup of ESBL-ST131 strains. A follow-up study based on whole-genome sequencing may be able to provide more clarity on this. The observed difference in mortality cannot be explained by a difference in the duration of follow up because it was only observed in the group that was positive upon entry in the study, and both groups (ESBL-ST131 and ESBL-non-ST131) have the same follow up duration.

Other studies that examined the duration of colonization for ESBL-ST131 compared to other ESBL-EC also found a longer duration of carriage for ESBL-ST131. Van Duijkeren et al. found that ESBL-EC colonization persisted for > 8 months in one-third of the ESBL-positive persons from the Dutch general population and found that longer colonization was statistically significant associated with the detection of phylogenetic group B2 and ST131, among others [14]. Titelman et al. included patients after a first-time ESBL-producing Enterobacteriaceae infection and showed that colonization with *E. coli* was still apparent after 12 months in 64% (n = 26), and 40% (n = 14) of those carrying *E. coli* ST131 or other STs, respectively (*p* = 0.12) [15]. Ismail et al. investigated the incidence and duration of colonization of ciprofloxacin-resistant *E. coli* in nursing homes in Michigan. The study showed that ST131 strains in residents were carried significantly longer when compared to non-ST131 strains (10 months versus 3 months) [16]. In all of the above studies, an ESBL positive culture (prevalence or clinical) was the starting point for inclusion in the studies, which could potentially influence the outcome of the studies.

In some studies on duration of colonization, ESBL-EC clearance is defined as two consecutive negative samples [17]. In this study, we defined ESBL-EC clearance when one sample no longer yielded ESBL-EC or when strain typing showed a different cluster type than found in the previous ESBL-EC positive culture of the resident. This approach was chosen to keep the results comparable to Overdevest et al. Furthermore, only a small proportion of the residents (2.7%) became ESBL-EC positive after a negative ESBL-EC culture. Moreover, using two negative cultures did not significantly alter the results.

This study has some limitations. First, there are several known risk factors for (prolonged) ESBL colonization duration, such as antibiotic use, proton pump inhibitor use or variables associated with higher need for care [14, 17]. Unfortunately, such clinical information was not acquired in the study and therefore we cannot further investigate the possible influence of (specific) resident factors.

Another limitation is the setting. The study was performed in a Dutch nursing home during an outbreak situation, which may reduce generalizability for other settings and/or patient populations. The major strengths of our study include the standardised cultures taken at defined intervals and most important the long follow-up period up to six years.

## Conclusion

A longer duration of colonization with ESBL-ST131 compared to ESBL-non-ST131 was found in the group who was ESBL-EC positive upon entering the study. In the subgroup of residents who acquired ESBL-EC during the study period, no difference in the duration of colonization between ESBL-ST131 and ESBL-non-ST131 was found. We conclude that the longer duration of colonization with ESBL-ST131 was caused by factors that occurred during the outbreak that preceded the observation period. In newly colonized individuals there was no difference in duration of colonization between ESBL-ST131 and other sequence types.

## Supplementary Information

**Additional file 1.** **Figure 1** **a-d** Schematic representation of determination colonisation duration in different situations whereby ‘loss of colonisation’ is reached with at least one sample no longer yielded ESBL-EC or when a sample showed a different cluster type than found in the previous ESBL-EC positive culture of the resident.

**Additional file 2.** **Figure 2** **a-e** Schematic representation of determination colonisation duration in different situations whereby ‘loss of colonisation’ is reached when at least two (instead of one) samples no longer yielded ESBL-EC or when two samples showed a different cluster type than found in the previous ESBL-EC positive culture of the resident.

**Additional file 3.** **Figure 3** Kaplan–Meier curve of ESBL-EC colonisation over time for all residents (n = 112) when considering a resident no longer colonised with *two* ESBL-EC negative culture/other strain type (p = 0.181).

**Additional file 4.** **Figure 4** Kaplan–Meier curve of ESBL-EC colonisation over time for residents who were ESBL-EC positive in their first prevalence survey the study (n = 55) when considering a resident no longer colonised with *two* ESBL-EC negative culture/other strain type (p = 0.101).

**Additional file 5.** **Figure 5** Kaplan–Meier curve of ESBL-EC colonisation over time for residents who acquired ESBL-EC during the study when considering a resident no longer colonised with *two* ESBL-EC negative culture/other strain type (p = 0.815).

**Additional file 6.** An overview of the proportion and reasons for events or censoring for the Kaplan-Meier survival analyses per selection group.

## Data Availability

The datasets used and/or analysed during the current study are available from the corresponding author on request.

## Abbreviations

AFLP: Amplified Fragment Length Polymorphism
CTX-M: Cefotaxime-hydrolyzing *β*-lactamase–Munich
EbSA: Extended-spectrum *β*-lactamase screening agar
*E. coli*: *Escherichia coli*
ESBL: Extended spectrum *β*-lactamase
ESBL-EC: Extended spectrum *β*-lactamase producing *E. coli*
ExPEC: Extraintestinal pathogenic *E. coli*
MALDI-TOF: Matrix-Assisted Laser Desorption-Ionisation-Time of Flight Mass Spectrometry
MLST: Multilocus sequence typing
SHV: Sulfhydryl variant of the TEM enzyme
SPSS: Statistical Package for the Social Sciences
ST: Sequence type
TEM: Temoneira class A extended-spectrum β-lactamase
TSB-VC: Tryptic soy broth containing cefotaxime and vancomycin
