# Supplementary figures and images for: Duration of rectal colonization with extended-spectrum beta-lactamase-producing Escherichia coli: results of an open, dynamic cohort study in Dutch nursing home residents (2013–2019)
Source: Antimicrob Resist Infect Control. 2022 Jul 15;11:98. doi: 10.1186/s13756-022-01132-9 (PMC9287922; doi:10.1186/s13756-022-01132-9)

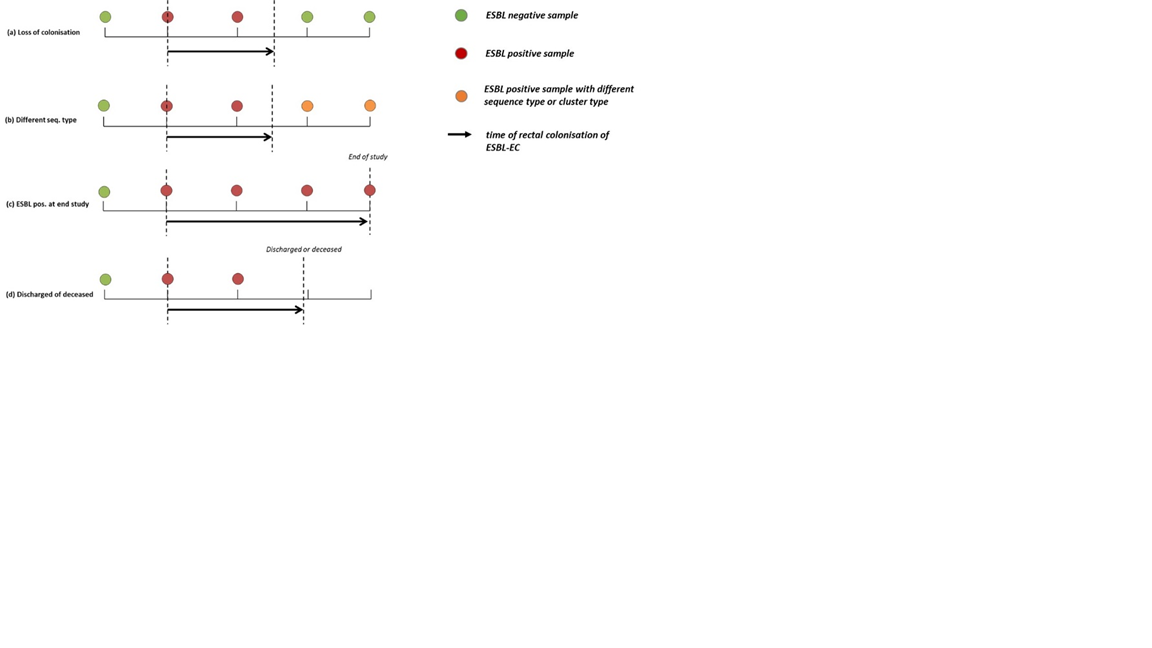

Supplement: Supplementary file 1 — Additional file 1. Figure 1 a-d Schematic representation of determination colonisation duration in different situations whereby ‘loss of colonisation’ is reached with at least one sample no longer yielded ESBL-EC or when a sample showed a different cluster type than found in the previous ESBL-EC positive culture of the resident. [file 13756_2022_1132_MOESM1_ESM.png]

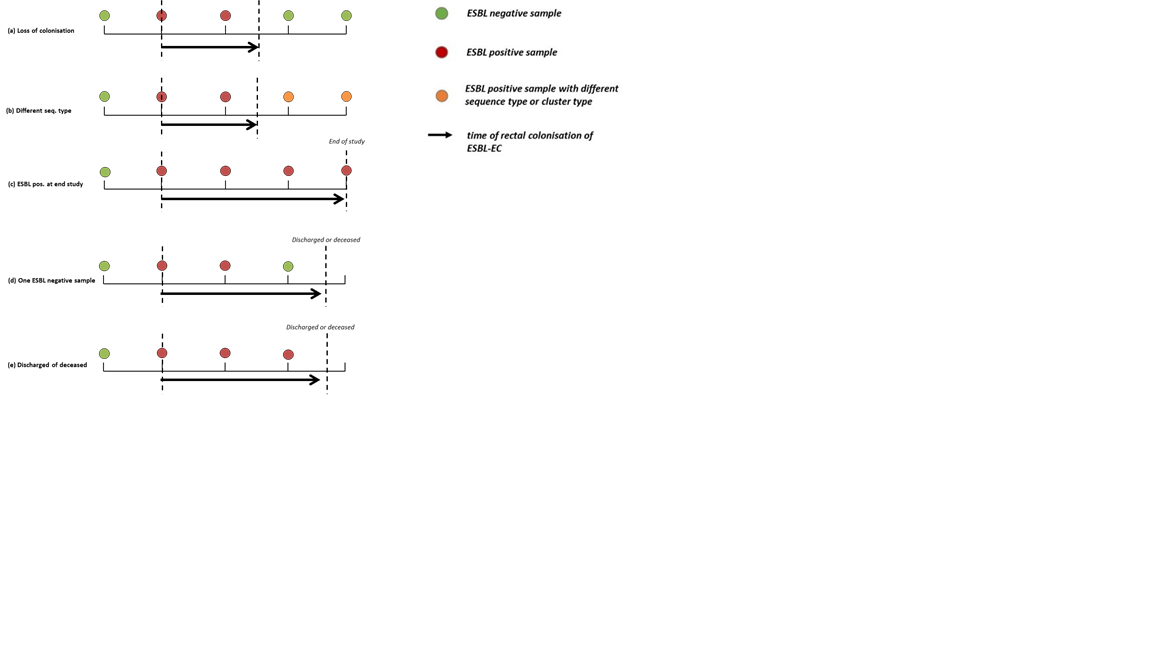

Supplement: Supplementary file 2 — Additional file 2. Figure 2 a-e Schematic representation of determination colonisation duration in different situations whereby ‘loss of colonisation’ is reached when at least two (instead of one) samples no longer yielded ESBL-EC or when two samples showed a different cluster type than found in the previous ESBL-EC positive culture of the resident. [file 13756_2022_1132_MOESM2_ESM.png]

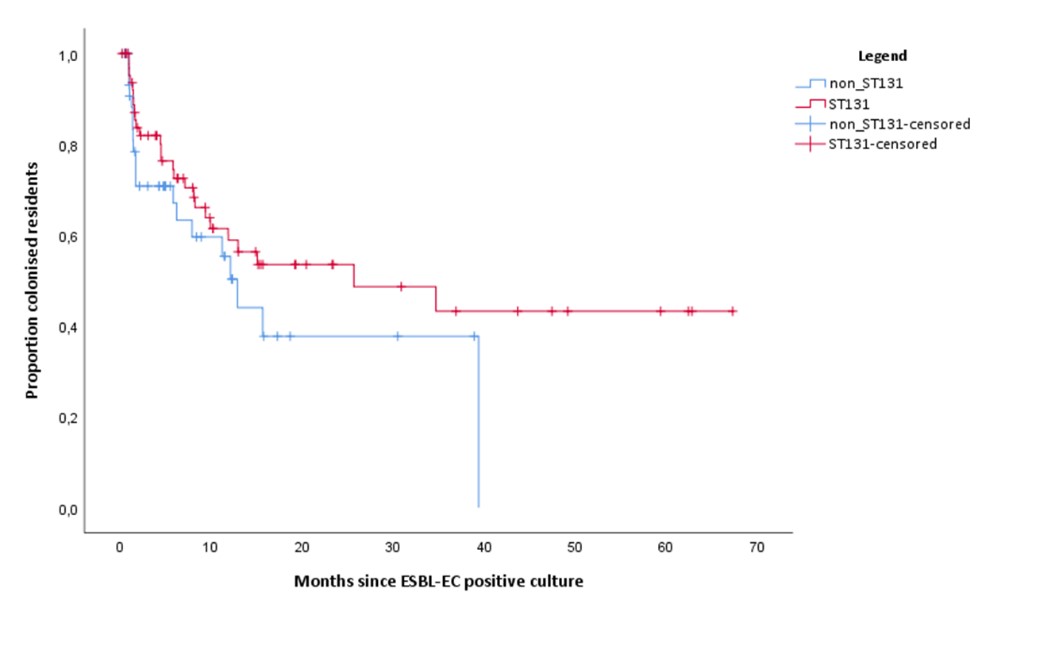

Supplement: Supplementary file 3 — Additional file 3. Figure 3 Kaplan–Meier curve of ESBL-EC colonisation over time for all residents (n = 112) when considering a resident no longer colonised with two ESBL-EC negative culture/other strain type (p = 0.181). [file 13756_2022_1132_MOESM3_ESM.jpg]

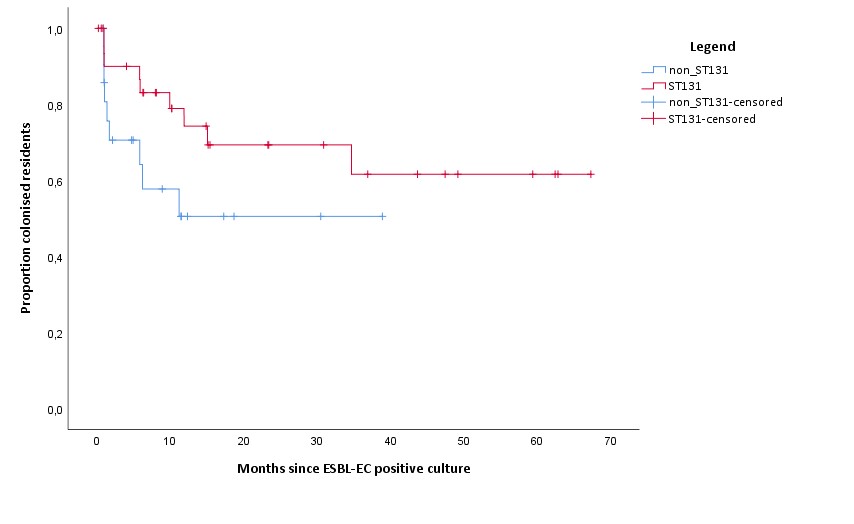

Supplement: Supplementary file 4 — Additional file 4. Figure 4 Kaplan–Meier curve of ESBL-EC colonisation over time for residents who were ESBL-EC positive in their first prevalence survey the study (n = 55) when considering a resident no longer colonised with two ESBL-EC negative culture/other strain type (p = 0.101). [file 13756_2022_1132_MOESM4_ESM.jpg]

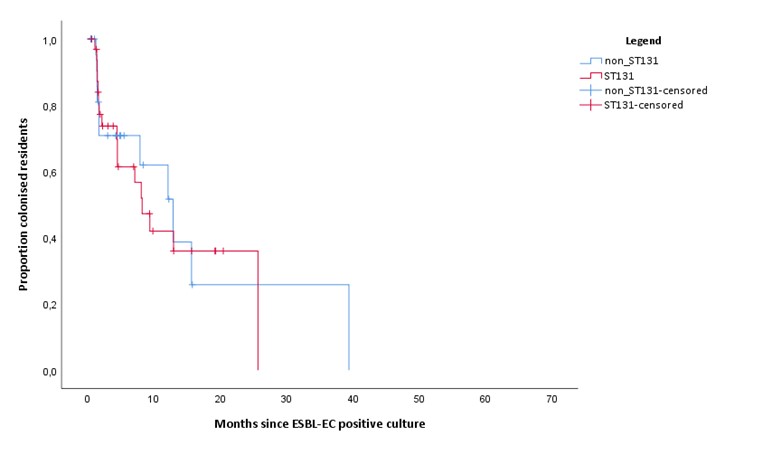

Supplement: Supplementary file 5 — Additional file 5. Figure 5 Kaplan–Meier curve of ESBL-EC colonisation over time for residents who acquired ESBL-EC during the study when considering a resident no longer colonised with two ESBL-EC negative culture/other strain type (p = 0.815). [file 13756_2022_1132_MOESM5_ESM.jpg]
