# Supplementary material for: Duration of rectal colonization with extended-spectrum beta-lactamase-producing Escherichia coli: results of an open, dynamic cohort study in Dutch nursing home residents (2013–2019)
Source: Antimicrob Resist Infect Control. 2022 Jul 15;11:98. doi: 10.1186/s13756-022-01132-9 (PMC9287922; doi:10.1186/s13756-022-01132-9)
Supplement: Supplementary file 6 — Additional file 6. An overview of the proportion and reasons for events or censoring for the Kaplan-Meier survival analyses per selection group. [file 13756_2022_1132_MOESM6_ESM.docx]

**Supplementary data**

**Supplementary table 1**: reasons for event or censoring in Kaplan Meier analysis for all residents with at least one ESBL-EC positive culture (n=112) - with **ONE** ESBL-EC negative cultures/other strain type to consider a resident no longer colonised.

| *Reasons for event of censoring in Kaplan Meier analysis* | **ESBL-ST131 (n=66)** | **ESBL-non-ST131 (n=46)** | ***p*** |
| --- | --- | --- | --- |
| Loss of colonisation (event), n (%) | 31 (47.0) | 22 (47.8) | *1.00* |
| Acquisition of other strain type (event), n (%) | 0 (0.0) | 4 (8.7) | *0.03* |
| Still ESBL-EC positive at end of study (censored), n (%) | 2 (3.0) | 0 (0.0) | *0.51* |
| Discharged in study period (censored), n (%) | 2 (3.0) | 5 (10.9) | *0.12* |
| Deceased in study period (censored), n (%) | 31 (47.0) | 15 (32.6) | *0.17* |

**Supplementary table 2**: reasons for event or censoring in Kaplan Meier analysis for residents who were ESBL-EC positive in their first prevalence survey in the study (n = 55) - with **ONE** ESBL-EC negative culture/other strain type to consider a resident no longer colonised.

| *Reasons for event of censoring in Kaplan Meier analysis* | **ESBL-ST131 (n=33)** | **ESBL-non-ST131 (n = 22)** | ***p*** |
| --- | --- | --- | --- |
| Loss of colonisation (event), n (%) | 11 (33.3) | 9 (40.9) | *0.58* |
| Acquisition of other strain type (event), n (%) | 0 (0.0) | 2 (9.1) | *0.16* |
| Still ESBL-EC positive at end of study (censored), n (%) | 1 (3,0) | 0 (0.0) | *1.00* |
| Discharged in study period (censored), n (%) | 1 (3,0) | 4 (18.2) | *0.14* |
| Deceased in study period (censored), n (%) | 20 (60.6) | 7 (31.8) | *0.05* |

**Supplementary table 3**: reasons for event or censoring in Kaplan Meier analysis for residents who acquired ESBL-EC colonisation during the study (n=57) - with **ONE** ESBL-EC negative culture/other strain type to consider a resident no longer colonised.

| *Reasons for event of censoring in Kaplan Meier analysis* | **ESBL-ST131 (n=33)** | **ESBL-non-ST131 (n=24)** | ***P*** |
| --- | --- | --- | --- |
| Loss of colonisation (event), n (%) | 20 (60.6) | 13 (54.2) | *0.79* |
| Acquisition of other strain type (event), n (%) | 0 (0.0) | 2 (8.3) | *0.17* |
| Still ESBL-EC positive at end of study (censored), n (%) | 1 (3.0) | 0 (0.0) | *1.00* |
| Discharged in study period (censored), n (%) | 1 (3.0) | 1 (4.2) | *1.00* |
| Deceased in study period (censored), n (%) | 11 (33.3) | 8 (33.3) | *1.00* |

**Supplementary table 4**: reasons for event or censoring in Kaplan Meier analysis for all residents with at least one ESBL-EC positive culture (n=112) - with **TWO** ESBL-EC negative cultures/other strain type to consider a resident no longer colonised.

| *Reasons for event of censoring in Kaplan Meier analysis* | **ESBL-ST131 (n=66)** | **ESBL-non-ST131 (n=46)** | ***p*** |
| --- | --- | --- | --- |
| Loss of colonisation (event), n (%) | 26 (39.4) | 19 (41.3) | *0.84* |
| Acquisition of other strain type (event), n (%) | 0 (0.0) | 1 (2.2) | *0.41* |
| Still ESBL-EC positive at end of study (censored), n (%) | 2 (3.0) | 0 (0.0) | *0.51* |
| Discharged in study period (censored), n (%) | 2 (3.0) | 5 (10.9) | *0.12* |
| Deceased in study period (censored), n (%) | 31 (47.0) | 15 (32.6) | *0.11* |
| Only one negative culture/culture with other strain type (censored), n (%) | 5 (7.6) | 6 (13.0) | *0.35* |

**Supplementary table 5**: reasons for event or censoring in Kaplan Meier analysis for residents who were ESBL-EC positive in their first prevalence survey in the study (n = 55) - with **TWO** ESBL-EC negative culture/other strain type to consider a resident no longer colonised.

| *Reasons for event of censoring in Kaplan Meier analysis* | **ESBL-ST131 (n=33)** | **ESBL-non-ST131 (n=22)** | ***p*** |
| --- | --- | --- | --- |
| Loss of colonisation (event), n (%) | 9 (27.3) | 9 (40.9) | *0.38* |
| Acquisition of other strain type (event), n (%) | 0 (0.0) | 0 (0.0) | *1.00* |
| Still ESBL-EC positive at end of study (censored), n (%) | 1 (3.0) | 0 (0.0) | *1.00* |
| Discharged in study period (censored), n (%) | 1 (3.0) | 4 (18.2) | *0.14* |
| Deceased in study period (censored), n (%) | 20 (60.6) | 7 (31.8) | *0.05* |
| Only one negative culture/culture with other strain type (censored), n (%) | 2 (6.1) | 2 (9.1) | *1.00* |

**Supplementary table 6**: reasons for event or censoring in Kaplan Meier analysis for residents who acquired ESBL-EC colonisation during the study (n=57) - with **TWO** ESBL-EC negative culture/other strain type to consider a resident no longer colonised.

| *Reasons for event of censoring in Kaplan Meier analysis* | **ESBL-ST131 (n=33)** | **ESBL-non-ST131 (n=24)** | ***p*** |
| --- | --- | --- | --- |
| Loss of colonisation (event), n (%) | 17 (51.5) | 10 (41.7) | *0.66* |
| Acquisition of other strain type (event), n (%) | 0 (0.0) | 1 (4.2) | *0.41* |
| Still ESBL-EC positive at end of study (censored), n (%) | 1 (3.0) | 0 (0.0) | *1.00* |
| Discharged in study period (censored), n (%) | 1 (3.0) | 1 (4.2) | *1.00* |
| Deceased in study period (censored), n (%) | 11 (33.3) | 8 (33.3) | *1.00* |
| Only one negative culture/culture with other strain type (censored), n (%) | 3 (9.1) | 4 (16.7) | *0.44* |
